# Integrating Single‐Cell Transcriptomics and Mendelian Randomization to Identify RAC1 as a Causal Metabolic Driver of Pericyte Dysfunction in Systemic Sclerosis

**DOI:** 10.1155/mi/6166654

**Published:** 2026-06-16

**Authors:** Xiaoqing Wang, Zheng Zhang, Enze Jiang, Mengdi Zhang, Guanglei Hu

**Affiliations:** ^1^ Department of Dermatology, Shanghai Ninth People’s Hospital, Shanghai Jiao Tong University School of Medicine, Shanghai, China, shsmu.edu.cn; ^2^ Orthopedic Department (No.1), Jing’an District Zhabei Central Hospital, Shanghai, China; ^3^ Department of General Practice, Puxing Community Health Service Center, Pudong New Area, Shanghai, China; ^4^ School of Life Sciences, Westlake University, Hangzhou, China, westlake.edu.cn

## Abstract

**Background:**

Systemic sclerosis (SSc) is an autoimmune disease characterized by vascular injury and progressive fibrosis. Although microvascular injury is an inciting event and pericytes are recognized as a major source of myofibroblasts, the precise phenotypic heterogeneity of pericytes in the SSc microenvironment and the genetic mechanisms driving their pathological transition remain elusive.

**Methods:**

This study systematically explored the cellular and genetic basis of pericyte dysfunction by integrating single‐cell RNA sequencing (scRNA‐seq) data from SSc patients with genome‐wide association study (GWAS) data using bidirectional Mendelian randomization (MR) analysis. Pseudotime trajectory analysis was used to reconstruct developmental lineages, while cell–cell communication and metabolic pathway analyses were conducted to uncover underlying mechanisms. Multiomics validation was performed using external bulk RNA‐seq datasets.

**Results:**

Single‐cell analysis revealed significant heterogeneity in pericyte subpopulations, specifically identifying a marked expansion of progenitor‐like pericytes, which were positioned at the root of the differentiation trajectory toward fibrotic phenotypes. Bidirectional MR analysis identified *RAC1* as a significant causal risk factor for SSc (OR = 2.0756, *p*  = 0.0046). Mechanistically, *RAC1*‐positive pericytes exhibited enhanced proinflammatory crosstalk with macrophages via the MIF‐(CD74 + CD44) signaling axis. Furthermore, these activated pericytes displayed distinct metabolic reprogramming, characterized by the upregulation of riboflavin and thiamine metabolism to support their bioenergetic demands. Transcriptomic validation further confirmed the aberrant overexpression of *RAC1* in SSc tissues.

**Conclusion:**

This study establishes a mechanistic link between *RAC1*‐mediated activation of progenitor‐like pericytes and SSc pathogenesis. *RAC1* acts as a causal driver promoting pathological pericyte transition and orchestrates a proinflammatory microenvironment through metabolic reprogramming and immune recruitment, offering a novel therapeutic target for SSc.

## 1. Introduction

Systemic sclerosis (SSc) is a heterogeneous autoimmune rheumatic condition characterized by widespread vascular abnormalities, immune system dysregulation, and progressive fibrosis of the skin and internal organs, leading to persistent and often irreversible organ dysfunction [1, 2]. The escalating socioeconomic strain on global healthcare systems is significantly driven by SSc, a condition that currently exhibits the highest disease‐specific mortality rate among all connective tissue disorders [1]. Epidemiological studies indicate that the global prevalence of SSc varies geographically, ranging from 7 to 489 cases per million individuals, with a striking female predominance [3]. Despite advances in management, the 10‐year survival rate remains suboptimal, hovering around 60%–80%, with deaths primarily driven by pulmonary fibrosis and pulmonary arterial hypertension [4]. While genetic predisposition is a key risk factor, environmental triggers such as silica exposure and organic solvents also contribute to the disease burden [5].

Extensive research evidence has shown that microvascular injury is the earliest inciting event in SSc pathogenesis, preceding the widespread fibrotic cascade [6]. Notably, pericytes—mural cells that encircle endothelial cells—may serve as a critical bridge between these vascular insults and tissue fibrosis [7]. Under pathological conditions, pericytes can detach from capillaries and transdifferentiate into myofibroblasts, a process known as the pericyte‐to‐myofibroblast transition (PMT), which has been widely recognized and supported in various fibrotic models [8]. The dysregulation of pericytes, particularly involving specific progenitor‐like subpopulations, is closely related to the progression of vascular rarefaction and collagen deposition [9]. The complex interaction of pericytes with immune cells (such as macrophages) and stromal cells can not only promote vascular instability but also participate in the proinflammatory immune response [10]. Consequently, the phenotypic dysregulation of pericytes is intimately associated with the onset, progression, and ultimate clinical outcomes of SSc.

Recently, the extensive application of single‐cell RNA sequencing (scRNA‐seq) and genome‐wide association study (GWAS) has significantly advanced our understanding of the intricate genetic architectures underlying multifactorial diseases, particularly by pinpointing pathogenic cell subsets and their transcriptomic dysregulation [11]. Research via single‐cell sequencing in SSc has revealed profound heterogeneity in fibroblasts and endothelial cells, indicating specific transcriptomic signatures associated with skin and lung fibrosis [11]. Moreover, GWAS has identified multiple risk loci associated with innate and adaptive immunity [12]. However, the application of these high‐throughput technologies to specifically dissect the pericyte compartment remains limited.

Although observational studies frequently implicate pericyte dysfunction in SSc fibrogenesis, the fundamental causal pathways and molecular architectures governing pericyte activation are still poorly understood. Bridging this knowledge gap requires the rigorous integration of single‐cell and genomic profiles to delineate how specific pericyte phenotypes dictate SSc pathogenesis, thereby facilitating the discovery of early clinical targets. Most existing studies rely on observational correlations, which are susceptible to confounding factors and reverse causation, failing to pinpoint the initiating genetic drivers.

Leveraging both single‐cell transcriptomics and bidirectional Mendelian randomization (MR), this work delineates the genetic and cellular underpinnings of SSc‐associated pericyte dysregulation. Specifically, a comparative assessment of sequencing profiles between SSc cohorts and healthy subjects allowed us to map compositional shifts within pericyte compartments, uncovering a distinct proliferative expansion of the progenitor‐like pericyte subset. We performed intercellular communication analysis to decode their crosstalk with the immune microenvironment and conducted in‐depth differential gene expression analysis. In addition, pseudotime trajectory analysis was also used to reveal the dynamic transcriptional changes of key genes during the differentiation from progenitor‐like states to activated fibrotic phenotypes. Integrating expression quantitative trait loci (eQTL) instruments with comprehensive SSc GWAS summary statistics, we deployed a bidirectional MR framework to pinpoint genes exerting causal influences on disease susceptibility. Subsequently, to substantiate the biological relevance of these nominated targets, we cross‐validated our findings against independent bulk transcriptomic cohorts, confirming the dysregulated transcription of these risk loci across peripheral tissues. Further analysis identified the key role of the *RAC1* gene, validating its causal link to SSc risk and elucidating its downstream effects on metabolic reprogramming and inflammatory signaling. Ultimately, our integrated approach elucidated the core transcriptional networks driving pericyte‐dependent SSc progression, thereby offering novel translational insights into diagnosing and managing this highly intractable condition.

## 2. Materials and Methods

### 2.1. Data Acquisition and Processing of scRNA‐seq Data

Raw scRNA‐seq data were obtained from the Gene Expression Omnibus (GEO) database under Accession Number GSE138669 [13]. The dataset comprised 22 samples, consisting of 12 samples from patients with SSc and 10 samples from healthy controls. Data processing was conducted using the Seurat package (v4.4.0) in *R* [14]. Quality control was rigorously applied by excluding cells meeting the following criteria: (1) fewer than 200 or more than 4000 detected genes (nFeature_RNA); (2) mitochondrial gene expression exceeding 10% (percent.mt). To mitigate the impact of multiplet captures and ensure analytical precision, DoubletFinder was applied to identify and remove doublet artifacts before downstream analysis. The filtered data were normalized using the LogNormalize method with a scale factor of 10,000. The top 2000 highly variable features were identified via the variance stabilizing transformation (vst) method. To eliminate batch effects associated with different samples, the Harmony algorithm was employed [15]. Dimensionality reduction was performed using PCA, and the first 10 principal components (PCs) were selected for Uniform Manifold Approximation and Projection (UMAP) visualization and downstream clustering.

### 2.2. Cell Clustering and Annotation

Unsupervised clustering was performed using the Louvain algorithm based on the Harmony‐integrated reductions. Cellular identities were assigned through careful manual curation, guided strictly by the expression profiles of established lineage‐specific markers, such as KRT1/5 (keratinocytes), COL1A1/DCN (fibroblasts), and RGS5/PDGFRB (pericytes). Specifically, the pericyte cluster was extracted for independent subclustering analysis. This subpopulation underwent a second round of variable feature selection, PCA, and Harmony integration. Five distinct pericyte phenotypes were identified: resting pericytes, progenitor‐like pericytes, fibrosis‐associated pericytes, myofibroblast‐like pericytes, and activated pericytes.

### 2.3. Pseudotime Trajectory and Gene Switch Analysis

To reconstruct the developmental lineage of pericytes, pseudotime trajectory analysis was performed using the Slingshot package [16]. The trajectory was inferred based on the UMAP embeddings, with progenitor‐like pericytes defined as the root state. To provide objective computational validation for this biological assumption, CytoTRACE (Cellular [Cyto] Trajectory Reconstruction Analysis using gene Counts and Expression) was utilized to estimate the differentiation potential and stemness of the pericyte subclusters. To identify key regulatory genes driving these transitions, the GeneSwitches package was utilized. Gene expression data were binarized (cutoff = 0.2), and a logistic regression model was applied to characterize the “switching” behavior of genes along the pseudotime. Genes with high model fitting quality were identified as key drivers, distinguishing between “on‐switching” (upregulated) and “off‐switching” (downregulated) patterns.

### 2.4. Cell–Cell Communication Analysis

Intercellular communication networks were inferred using the CellChat package, focusing on the “Secreted Signaling” database [17]. Ligand‐receptor interactions were identified based on the expression of genes in the progenitor‐like pericyte subpopulation and other cell types. Interactions involving fewer than 10 cells were filtered out to ensure robustness. Significant signaling pathways were visualized using circle plots and bubble plots, highlighting the signaling strength mediated by the pericyte subsets.

### 2.5. Bidirectional MR Analysis

To systematically investigate the causal directionality between pericyte‐derived marker genes and SSc, a bidirectional two‐sample MR analysis was designed.

Forward MR analysis: Key marker genes (e.g., *RAC1* and *PSMB9*) identified from the single‐cell analysis served as exposures. Instrumental variables (IVs) were selected from eQTL data (sourced from the eQTLGen consortium database utilizing whole blood eQTLs) based on genome‐wide significance (*p* < 5e–08, *F*‐statistic > 10) and independence (*r* ^2^ < 0.001). Summary statistics for SSc were obtained from the FinnGen consortium (ID: finn‐b‐M13_SYSTSLCE). The inverse variance weighted (IVW) method was employed as the primary analytical tool [18], complemented by MR‐Egger and weighted median methods. Statistical significance for the MR causal estimates was reported using nominal *p*‐values. Given the targeted nature of our MR analysis—focusing only on a limited set of a priori candidates derived from scRNA‐seq—and the orthogonal multiomics validation provided, multiple testing correction (e.g., FDR) was not strictly applied to avoid inflating Type II errors, though we explicitly denote these as nominal values. Sensitivity analyses, including Cochran’s *Q* test for heterogeneity and the MR‐Egger intercept test for pleiotropy, were conducted to validate the causal inference [19, 20].

Reverse MR analysis: To exclude reverse causation, SSc was treated as the exposure, and the expression levels of the candidate genes were treated as the outcome. Genetic variants associated with SSc were selected as IVs, and their effects on gene expression were assessed using the same MR framework. A nonsignificant result in the reverse MR analysis was considered supportive evidence for the directionality of the causal link from gene expression to disease risk.

### 2.6. Bulk RNA‐seq Validation, Clinical Correlation, and Connectivity Map (CMap) Analysis

To cross‐validate the single‐cell findings at the tissue level, bulk RNA‐seq data were retrieved from GSE130955. Raw counts were converted to transcripts per million (TPM) and log_2_‐transformed [21]. The expression levels of key risk genes (e.g., RAC1) were compared between SSc patients and healthy controls using the Wilcoxon rank‐sum test. To ensure that bulk tissue upregulation was not merely an artifact of altered cellularity, the CIBERSORTx deconvolution algorithm was applied to estimate the absolute abundance of progenitor‐like pericytes. Pearson correlation analysis was subsequently performed to assess the clinical relevance of RAC1 expression against the modified Rodnan skin score (mRSS). Furthermore, to identify actionable pharmacological interventions, DEGs distinguishing RAC1+ from RAC1− pericytes were queried against the CMap and DSigDB databases to retrieve FDA‐approved compounds capable of reversing this pathogenic signature. All statistical analyses were performed in R software. A *p*‐value < 0.05 was considered statistically significant.

## 3. Results

### 3.1. Single‐Cell Transcriptional Landscape and Cellular Heterogeneity Identification

**Figure 1:**
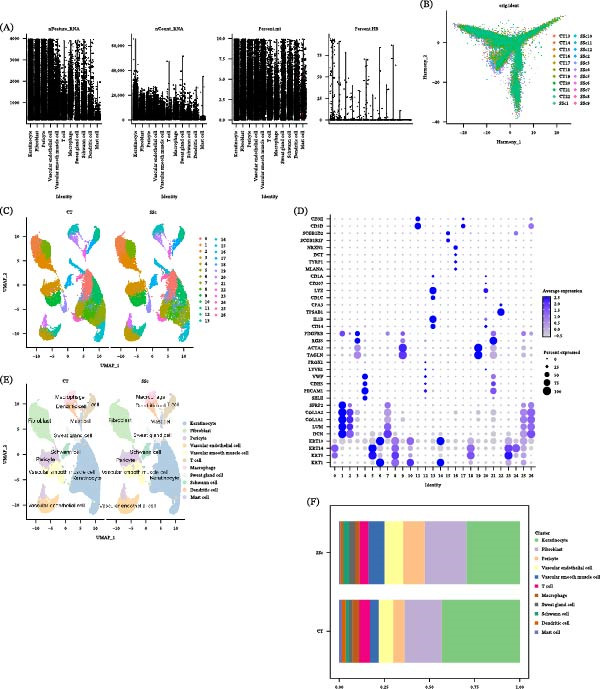
Single‐cell transcriptomic landscape and cellular heterogeneity in systemic sclerosis (SSc). (A) Quality control metrics visualized by violin plots, displaying the number of detected features (nFeature_RNA), unique molecular identifier counts (nCount_RNA), mitochondrial gene percentage (percent.mt), and hemoglobin gene percentage (percent.HB) across different cell types. (B) Scatter plot of Harmony‐integrated data. (C) UMAP plot showing the dimensionality reduction and unsupervised clustering results, identifying 27 distinct cell clusters. (D) Annotated UMAP visualization of 11 major cell lineages identified based on canonical marker genes. (E) Dot plot illustrating the expression patterns of top marker genes for each cell type. (F) Stacked bar plot displaying the proportional distribution of identified cell types across the different clusters.

To guarantee the robustness of subsequent evaluations, the raw scRNA‐seq datasets underwent rigorous initial filtering to eliminate substandard cells and poorly expressed transcripts. As depicted in Figure 1A, we mitigated technical artifacts by applying strict quality control thresholds based on the diversity of expressed genes (nFeature_RNA), total unique molecular identifiers (nCount_RNA), and the mitochondrial transcript ratio (percent.mt). Subsequently, PCA was utilized to extract the main biological information and reduce the dimensionality of the high‐dimensional dataset. To eliminate potential batch effects between the CT and SSc groups, the Harmony algorithm was applied. As depicted in Figure 1B, the applied integration strategy successfully aligned datasets across multiple origins, largely eliminating observable technical batch variations. Subsequent unsupervised clustering and nonlinear dimensionality reduction via UMAP partitioned this harmonized dataset into 27 unique cellular subsets (Figure 1C). By identifying significantly differentially expressed genes and aligning them with canonical marker genes reported in the literature, these clusters were annotated into 11 major cell lineages (Figure 1D). The expression patterns of specific marker genes used for annotation were visualized using a dot plot (Figure 1E). Notably, keratinocytes were characterized by the high expression of KRT1, KRT5, and KRT14; fibroblasts exhibited significant expression of COL1A1, COL1A2, and DCN; while T cells were identified based on the expression of CD3D and CD3E. Other identified cell types included vascular endothelial cells (PECAM1, CDH5), pericytes (RGS5, PDGFRB), macrophages (LYZ, CD14), mast cells (TPSAB1, CPA3), as well as dendritic cells, Schwann cells, sweat gland cells, and vascular smooth muscle cells. Finally, the proportional distribution of these cell subpopulations across the identified clusters was analyzed (Figure 1F), revealing the cellular heterogeneity and the complex composition of the tissue microenvironment under the studied conditions.

### 3.2. Subpopulation Identification and Functional Characterization of Pericytes

**Figure 2:**
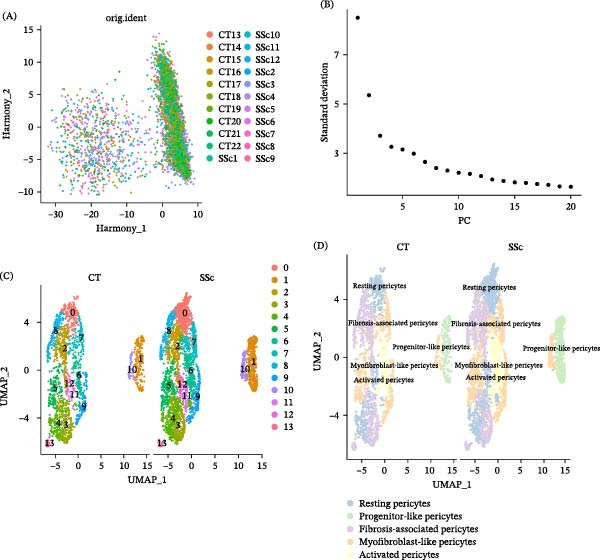
Subpopulation identification and functional characterization of pericytes in SSc. (A) Scatter plot of Harmony‐integrated data, demonstrating the effective removal of batch effects and integration of samples from CT and SSc groups. (B) Elbow plot illustrating the standard deviation of PCs, used to determine the optimal number of PCs for downstream clustering. (C) UMAP plots showing the dimensionality reduction and unsupervised clustering results, identifying 14 distinct pericyte subclusters split by condition. (D) Annotated UMAP visualization of the five identified pericyte functional phenotypes based on specific marker gene expression profiles.

To further elucidate the heterogeneity and potential pathological roles of pericytes in the disease microenvironment, the pericyte subpopulation was extracted from the global cellular landscape for in‐depth downstream analysis. As illustrated in Figure 2A, the Harmony integration framework was implemented to systematically neutralize intersample technical variations. The visualization confirms that samples from the control (CT) and disease (SSc) groups were effectively integrated, eliminating technical variations while preserving biological heterogeneity. Dimensionality reduction was performed using PCA. Based on the elbow plot (Figure 2B), the inflection point was utilized to determine the optimal number of PCs for subsequent clustering analyses, ensuring that the majority of the variation was captured. Utilizing UMAP for nonlinear dimensionality reduction, we identified 14 distinct pericyte subclusters (Figure 2C). By analyzing specific marker gene expression profiles and referencing established cell states in the literature, these subclusters were annotated into five distinct functional phenotypes (Figure 2D). These included resting pericytes, progenitor‐like pericytes, fibrosis‐associated pericytes, myofibroblast‐like pericytes, and activated pericytes. Notably, a visual comparison between the CT and SSc groups in the UMAP space suggests potential shifts in the abundance or state of these subtypes under pathological conditions, particularly within the fibrosis‐associated and activated subpopulations.

### 3.3. Expansion and Functional Trajectory of Progenitor‐Like Pericytes in SSc

**Figure 3:**
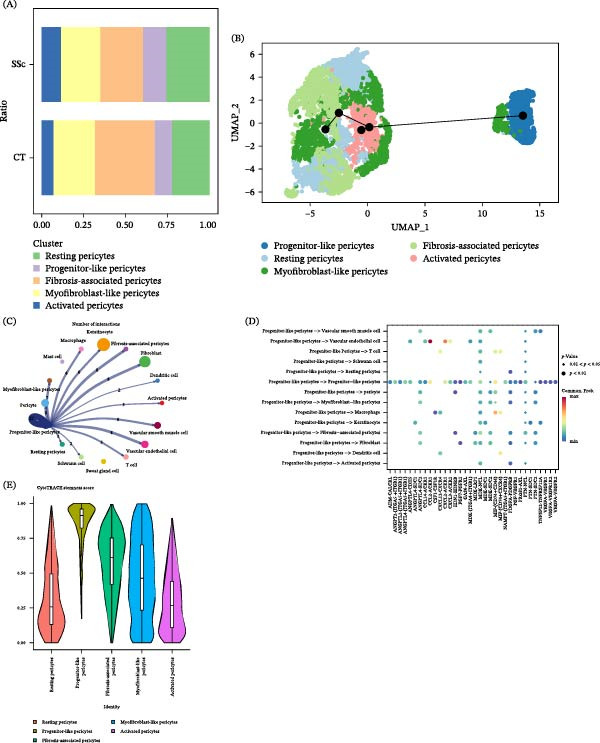
Expansion and intercellular communication of progenitor‐like pericytes. (A) Stacked bar plot displaying the proportional distribution of pericyte subpopulations in CT and SSc groups, highlighting the marked expansion of progenitor‐like pericytes in the disease state. (B) Pseudotime trajectory analysis projected onto the UMAP space, illustrating the developmental lineage where progenitor‐like pericytes serve as the root differentiating toward fibrotic phenotypes. (C) Circle plot visualizing the intercellular communication network, showing the number of significant interactions between progenitor‐like pericytes and other cell types. (D) Dot plot characterizing specific ligand‐receptor signaling pathways, such as VEGFA‐VEGFR and MIF axes, mediated by progenitor‐like pericytes. (E) Violin plot displaying the CytoTRACE stemness scores across different pericyte subclusters, computationally validating that progenitor‐like pericytes possess the highest differentiation potential.

Given the observation that progenitor‐like pericytes were significantly enriched in the disease group, this specific subpopulation was prioritized for further investigation to uncover its dynamic role in disease progression. As illustrated in the proportional analysis (Figure 3A), a dramatic shift in cellular composition was observed: while resting pericytes were predominant in the CT group, the SSc group was characterized by a marked expansion of progenitor‐like pericytes and fibrosis‐associated pericytes, suggesting a pathological transition from a quiescent to an activated, proliferative state. Consistent with this biological assumption, CytoTRACE analysis computationally confirmed that the progenitor‐like pericytes possessed the highest stemness score among all identified subclusters (Figure 3E), strongly solidifying their assignment as the developmental root state. To delineate the developmental relationships and potential differentiation pathways of these cells, pseudotime trajectory analysis was performed (Figure 3B). The trajectory inferred a developmental continuum where progenitor‐like pericytes were positioned at the root, potentially serving as a reservoir that differentiates into downstream effectors, including fibrosis‐associated pericytes and activated pericytes. This directional differentiation suggests that the expanded progenitor‐like population may drive the accumulation of fibrotic subtypes in the SSc microenvironment. To explore the molecular mechanisms governing the crosstalk between progenitor‐like Pericytes and other cell types, intercellular communication analysis was conducted. The interaction network (Figure 3C) revealed that progenitor‐like pericytes established extensive communication links, particularly with vascular endothelial cells, vascular smooth muscle cells, and fibroblasts. Further dissection of ligand‐receptor pairs via dot plot visualization (Figure 3D) identified key signaling axes. Notably, progenitor‐like pericytes were found to communicate with endothelial cells through angiogenic pathways such as VEGFA‐VEGFR and ANGPTL‐ITGA5/ITGB1. Furthermore, significant interactions with immune cells (macrophages and dendritic cells) were mediated by the MIF‐(CD74+CD44/CXCR4) axis, while the MDK signaling family (e.g., MDK‐NCL and MDK‐SDC1) appeared to be a broad‐spectrum regulator involved in interactions with multiple cell types.

### 3.4. Causal Association Between Progenitor‐Like Pericyte Marker Genes and SSc

**Figure 4:**
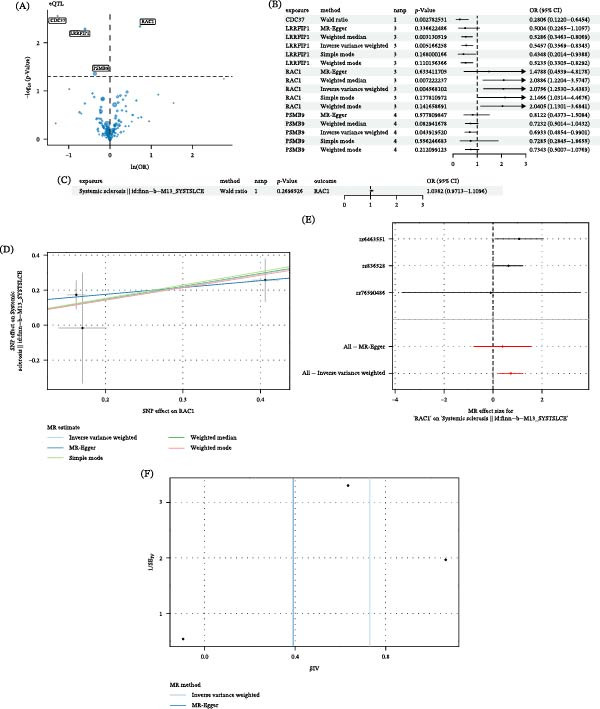
Mendelian randomization (MR) analysis of pericyte marker genes and SSc risk. (A) Volcano plot visualizing the selection of significant IVs for key pericyte marker genes based on genome‐wide significance thresholds. (B) Forest plot summarizing the bidirectional MR results, identifying RAC1 as a significant causal risk factor for SSc. (C) Reverse MR analysis table demonstrating the absence of a causal effect of SSc on RAC1 expression, effectively ruling out reverse causality. (D) Scatter plot illustrating the consistent positive correlation between RAC1‐associated genetic variants and SSc risk across multiple MR methods. (E) Forest plot displaying the individual effect sizes of valid SNPs used as instrumental variables for RAC1. (F) Funnel plot showing the symmetrical distribution of SNPs, indicating the absence of significant publication bias or horizontal pleiotropy.

To further validate the potential causal involvement of the expanded progenitor‐like pericyte subpopulation in the pathogenesis of SSc, a two‐sample MR analysis was conducted utilizing key marker genes identified from the single‐cell transcriptomic data as exposures. As visualized in the volcano plot (Figure 4A), significant IVs were selected based on genome‐wide significance thresholds to ensure the robustness of the causal inference. The results of the MR analysis, summarized in Figure 4B, revealed distinct causal relationships for specific marker genes. RAC1 was identified as a significant risk factor for SSc; the IVW method demonstrated that genetically predicted higher expression of RAC1 was associated with an increased risk of the disease (OR = 2.0756, 95% CI: 1.2530–3.4383, nominal *p* = 0.0046). This finding was corroborated by the weighted median method (OR = 2.0886, *p* = 0.0072), suggesting a robust positive correlation. In contrast, LRRFIP1, CDC37, and PSMB9 exhibited protective effects. Specifically, the IVW estimate for LRRFIP1 indicated a reduced risk of SSc (OR = 0.5457, 95% CI: 0.3569–0.8343, *p* = 0.0052), and CDC37 showed a similar protective trend utilizing the Wald ratio method (OR = 0.2806, *p* = 0.0028). Given the prominent risk effect of RAC1, it was prioritized for further sensitivity analysis. The scatter plot (Figure 4D) illustrated a consistent positive slope across different MR methods, reinforcing the linear relationship between RAC1 variants and the SSc risk. The forest plot of individual SNPs (Figure 4E) displayed that the effect sizes of valid IVs were generally distributed on the risk‐promoting side. Furthermore, the funnel plot (Figure 4F) showed a symmetrical distribution of SNPs, indicating the absence of significant publication bias or horizontal pleiotropy (Cochran’s *Q* test *p* = 0.452; MR‐Egger intercept test *p* = 0.613). Finally, to rule out reverse causality, a reverse MR analysis was performed (Figure 4C), which showed no significant causal effect of SSc on RAC1 expression (*p* = 0.270), thereby consolidating the conclusion that RAC1 may act as a driver in disease development.

### 3.5. Intercellular Crosstalk and Metabolic Reprogramming of RAC1+ Pericytes

To further elucidate the functional implications of RAC1 expression in pericytes, we stratified the population into RAC1+ and RAC1− subsets. The cellular interaction landscape, visualized in (Figure 5A), revealed that RAC1+IM (immunomodulatory) pericytes orchestrated a dense communication network, exhibiting particularly strong interactions with macrophages, fibroblasts, and vascular endothelial cells. Furthermore, network analysis of cellular interactions (Figure 5C) positioned progenitor‐like pericytes as a central signaling hub within the microenvironment. This analysis revealed extensive communication pathways, with progenitor‐like pericytes exhibiting the highest number of interactions with fibrosis‐associated pericytes, followed by substantial crosstalk with macrophages and fibroblasts. Detailed analysis of ligand‐receptor pairs (Figure 5B) highlighted that these interactions were predominantly mediated by proinflammatory chemokine signaling axes. Notably, RAC1+IM pericytes communicated with macrophages and dendritic cells via CCL2‐ACKR1, CXCL2‐ACKR1, and MIF‐(CD74+CD44) pathways and with endothelial cells via CXCL8‐ACKR1, suggesting a critical role in recruiting immune cells and modulating the inflammatory microenvironment. To reconstruct the transcriptional trajectory governing this specific phenotype, pseudotime analysis was performed based on binarized gene expression data (Figure 5B,D). The regression of gene expression over pseudotime identified key transcription factors and surface markers driving the RAC1+IM state. As depicted in Figure 5D, genes such as FOSL1, NR4A1, and NFATC2 were upregulated along the pseudo‐timeline, correlating with the expression trajectory of RAC1. Interestingly, the protective MR genes (LRRFIP1 and PSMB9) were also mapped onto this trajectory, providing a spatial reference for their potential regulatory antagonism against RAC1‐mediated pathways. Finally, pathway overrepresentation analysis indicated a putative metabolic shift within RAC1+IM pericytes relative to their RAC1− counterparts and other subclusters (Figure 5E). While resting pericytes were enriched in biotin metabolism, RAC1+IM pericytes exhibited significantly elevated transcriptomic signatures associated with riboflavin, thiamine, and retinol metabolism. While acknowledging the inherent limitations of transcriptomics‐based metabolic inference, these signatures collectively suggest that RAC1‐driven activation may require bespoke bioenergetic and biosynthetic support to sustain its proinflammatory and fibrotic functions.

Figure 5Intercellular crosstalk and metabolic reprogramming of RAC1+ pericytes. (A) Circle plot visualizing the intercellular interaction network of RAC1+ pericytes. (B) Histogram of gene expression distribution utilized for determining data binarization thresholds for downstream trajectory analysis. (C) Dot plot characterizing specific proinflammatory ligand‐receptor signaling pairs. (D) Scatter plot derived from GeneSwitches analysis, identifying key transcription factors and genes that are upregulated along the RAC1+IM pseudo‐timeline. (E) Dot plot displaying metabolic pathway enrichment.

**Figure:**
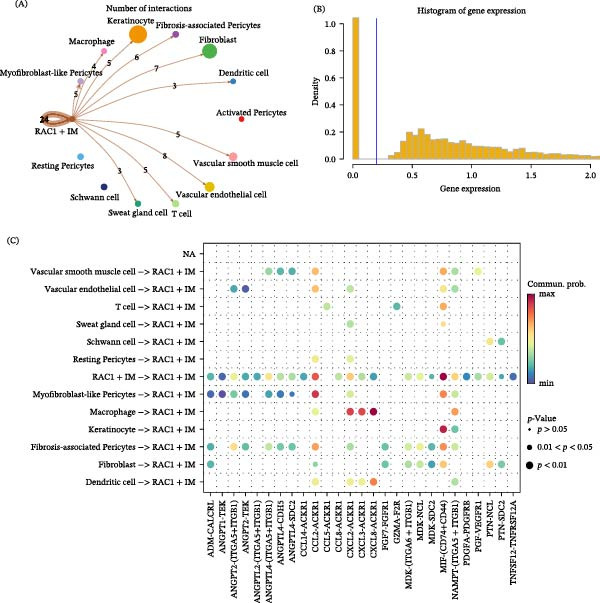

**Figure:**
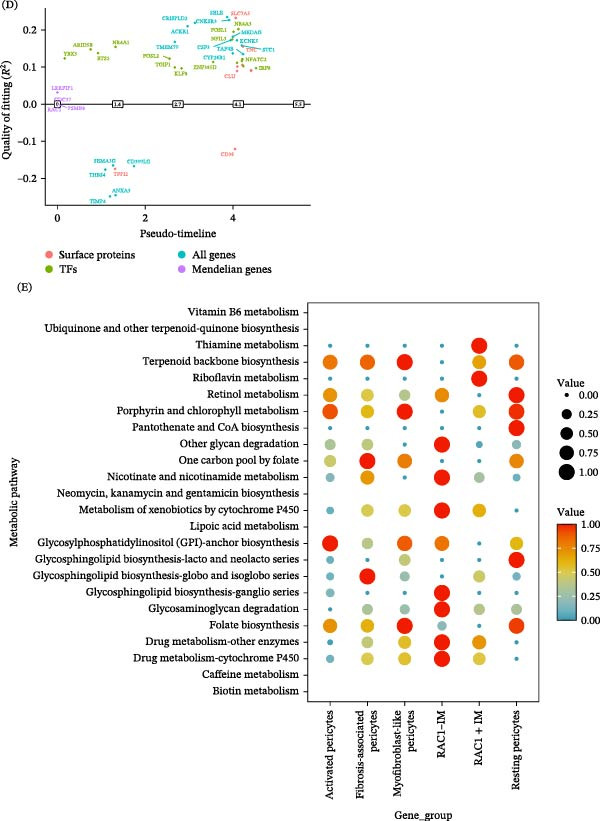

### 3.6. Transcriptional Validation and Expression Profiling of Key MR‐Identified Genes

To cross‐validate the findings from the MR analysis with the actual transcriptional landscape of the disease, the expression profiles of the identified causal genes (RAC1, CDC37, LRRFIP1, and PSMB9) were examined in the single‐cell dataset. As visualized in the dot plot (Figure 6A), these genes exhibited distinct cellular distribution patterns. Notably, RAC1 displayed robust and widespread expression across multiple lineages, including pericytes, fibroblasts, and macrophages, suggesting its potential function as a ubiquitous regulator in the stromal‐immune microenvironment. In contrast, PSMB9 showed a more restricted expression pattern, predominantly enriched in immune cell subsets such as macrophages, dendritic cells, and T cells. Focusing on the spatial distribution of the primary risk factor within the pericyte compartment, feature plots were generated (Figure 6B). The visualization confirmed that RAC1 was widely expressed across various pericyte phenotypes in both control (CT) and SSc conditions, maintaining its high expression levels, particularly in the activated and fibrosis‐associated clusters. To quantitatively assess the differential expression of these genes between health and disease, a heatmap was constructed (Figure 6C). The analysis revealed a clear transcriptomic shift, where the SSc group was characterized by a distinct upregulation of RAC1 compared to the healthy group. This observation was further statistically corroborated by box plot analysis (Figure 6D). The Wilcoxon rank‐sum test demonstrated that RAC1 expression was significantly elevated in SSc patients (*p* = 3.7e‐05). Conversely, highlighting the biological consistency of our findings, the causally protective genes exhibited a corresponding significant downregulation in the SSc single‐cell dataset compared to healthy controls (LRRFIP1, *p* = 0.0039; CDC37, *p* = 0.026) (Supporting Information 1: Figure S1). Furthermore, CIBERSORTx deconvolution of the bulk cohort confirmed a significantly higher estimated abundance of progenitor‐like pericytes in the RAC1‐high subgroup (*p* = 0.038) (Figure 6E), demonstrating that the bulk tissue upregulation of RAC1 is intimately linked to the expansion of this specific pericyte subset rather than mere broad immune infiltration. Crucially, extending these findings to clinical relevance, bulk RAC1 expression exhibited a significant positive correlation with the clinical severity index mRSS (*R* = 0.31, *p* = 0.023) (Figure 6F). Finally, CMap analysis successfully identified several FDA‐approved compounds capable of potentially reversing the RAC1+ profibrotic transcriptomic signature (Supporting Information 2: Table S1).

Figure 6Transcriptional validation and expression profiling of key MR‐identified genes. (A) Dot plot visualizing the cellular distribution patterns of identified causal genes across multiple cell lineages. (B) Feature plots illustrating the specific expression distribution of RAC1 within pericyte subpopulations in CT and SSc conditions. (C) Heatmap comparing the expression levels of the identified risk and protective genes between healthy controls and SSc patients. (D) Box plot statistically confirming the significant upregulation of RAC1 expression in SSc patients compared to healthy controls. (E) Bar plot showing the estimated relative abundance of progenitor‐like pericytes in *RAC1*‐high versus *RAC1*‐low SSc patient subgroups, derived from CIBERSORTx deconvolution of the bulk RNA‐seq cohort (GSE130955) (*p* = 0.038). (F) Scatter plot with a linear regression line revealing a significant positive correlation between bulk *RAC1* expression and the clinical severity index, modified Rodnan skin score, in SSc patients (*R* = 0.31, *p* = 0.023).

**Figure:**
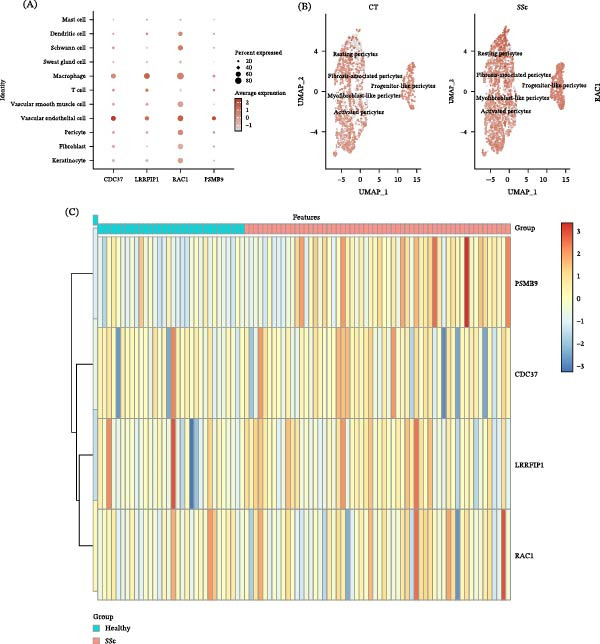

**Figure:**
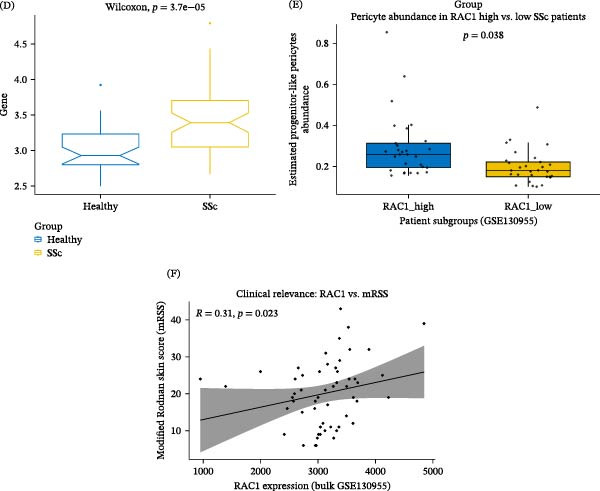

## 4. Discussion

SSc and its severe sequelae—most notably pulmonary fibrosis and pulmonary arterial hypertension—dramatically compromise patient well‐being while imposing a profound socioeconomic toll on global healthcare infrastructures. SSc is characterized by high cause‐specific mortality and substantial morbidity, often affecting individuals in the prime of their lives. Because the fibrovascular cascade dictates SSc pathogenesis—with aberrant pericyte activity emerging as a pivotal mediator—decoding the transcriptional regulation and phenotypic diversity of these cells is essential for advancing therapeutic interventions. Therefore, delineating these underlying networks carries profound translational value, offering promising new therapeutic vulnerabilities for this treatment‐resistant condition.

SSc involves a complex interplay of pathogenic factors, among which vascular injury and genetic susceptibility are two significant representatives. Similar to environmental insults in lung diseases, microvascular damage in SSc induces the release of a plethora of inflammatory mediators and profibrotic growth factors through oxidative stress pathways. In predisposed individuals, these primary triggers provoke a complex cascade encompassing genetic and epigenetic modifications, mitochondrial impairment, oxidative damage, and severe immune dysregulation. Collectively, this pathogenic network fuels SSc evolution, orchestrating the critical pathophysiological shift from early vascular inflammation to irreversible fibrogenesis. Additionally, epidemiological and experimental studies have shown that pericyte loss and dysfunction are common and important independent risk factors for the progression of tissue fibrosis. A tissue microenvironment conducive to massive collagen overproduction is primarily forged by a combination of unremitting inflammation and hypoxic oxidative stress during SSc pathogenesis. These localized stressors not only dismantle intrinsic vascular repair pathways but also trigger the pathological expansion and lineage transition of resident mural populations [22]. Furthermore, PMT serves as a critical mechanistic bridge linking vascular impairment to subsequent fibrotic remodeling, fundamentally driving the SSc‐associated vascular alterations and downstream organ dysfunction.

Recognizing that SSc patients universally display pronounced vascular fragility and amplified pericyte activation, the dynamic remodeling of the perivascular stroma presents crucial translational targets. Evaluating these pericyte‐specific shifts is therefore essential for mapping the trajectory from initial vascular insult to overt fibrosis; data from other fibrotic models demonstrate that inflammation mediated by specific pericyte subpopulations plays a crucial role in the development of myofibroblasts. Furthermore, it has been found that in patients with SSc, the pericyte population undergoes a phenotypic shift in both the skin and internal organs. Additionally, the presence of specific activated pericyte signatures is correlated with the disease severity and progression. The advent of single‐cell transcriptomics has enabled high‐resolution profiling of stromal heterogeneity, shedding light on the distinct transcriptional shifts that distinguish SSc cohorts from normotypic individuals. These studies reveal that inflammation‐induced plasticity may play a critical role during the early stages of SSc, especially concerning the regulatory imbalance of progenitor‐like cells. There is growing evidence that changes in pericytes, particularly the expansion of progenitor‐like pericytes, represent an important cellular link between microvascular damage and fibrosis. Decoding the interplay between vascular deterioration and fibrotic remodeling presents a critical avenue for elucidating their coevolution. Ultimately, translating these microenvironmental dynamics into clinical practice will significantly enhance early detection, therapeutic targeting, and the delivery of personalized SSc management.

Driven by the need to establish new theoretical and therapeutic paradigms for SSc, this study systematically explored how pericyte dysfunction influences disease susceptibility. Through differential expression profiling of scRNA‐seq data, we first pinpointed critical marker genes orchestrating the shift of pericytes from progenitor‐like to activated phenotypes. Subsequently, we executed bidirectional MR analyses—deploying eQTLs as genetic instruments—to decipher the causal impact of these specific genes on SSc risk. Notably, *RAC1* was identified as a potent pathogenic risk factor, exhibiting a strong causal association with SSc development (OR = 2.0756; 95% CI: 1.2530–3.4383; *p* = 0.0046). In stark contrast, *LRRFIP1* (OR = 0.5457; *p* = 0.0052) and *CDC37* (OR = 0.2806; *p* = 0.0028) demonstrated clear protective properties, illustrating a highly complex gene regulatory network governing pericyte‐mediated SSc.

The RAC1 gene, also known as Ras‐related C3 botulinum toxin substrate 1, encodes a GTPase belonging to the RAS superfamily of small GTP‐binding proteins. It consists of ~192 amino acids and is characterized by its ability to regulate the actin cytoskeleton [23]. Mapped to the 7p22.1 chromosomal locus, *RAC1* has emerged as a key pathogenic driver in diverse metastatic and fibrotic conditions. Notably, the transcriptional upregulation or functional hyperactivity of this gene drives multiple pathophysiological cascades—spanning oncogenic dissemination, aberrant wound repair, and pervasive tissue fibrosis [24, 25]. At the cellular level, such *RAC1* amplification actively facilitates the mobility, expansion, and phenotypic switching of mesenchymal populations. Furthermore, functioning as an essential regulatory subunit of the NADPH oxidase (NOX) holoenzyme, *RAC1* fundamentally orchestrates the synthesis of reactive oxygen species (ROS) [26]. Consequently, quantifying its expression offers a robust prognostic indicator for evaluating cellular hyperactivation and the severity of the oxidative burden, thereby facilitating the prediction of clinical trajectories and therapeutic efficacies. Presently, pharmacological strategies aimed at antagonizing the Rho/Rac GTPase cascade are under active evaluation, heralding a promising therapeutic frontier for the management of fibrovascular pathologies.

The transcriptomic and functional imbalance of cytoskeletal regulators, alongside their inflammatory mediators, critically dictates the clinical trajectory of SSc. Targeting these specific pathways thus presents a potent translational strategy to govern chronic inflammatory responses and vascular deterioration. Notably, contemporary studies have validated this approach, revealing that RAC1 inhibition significantly impairs myofibroblastic conversion and subsequent collagen deposition in a variety of experimental fibrotic models. As an illustration, the targeted pharmacological antagonism of the RAC1 cascade successfully mitigates dermal fibrogenesis within bleomycin‐challenged murine models. Such empirical data robustly substantiate the clinical viability of RAC1 inhibition, underscoring its immense translational promise for SSc management. Furthermore, given that RAC1 integrates signals from various growth factors (such as TGF‐β and PDGF) to drive cytoskeletal reorganization, targeting this central node could offer a more potent therapeutic effect than inhibiting single upstream receptors.

However, the protective effects observed for genes like LRRFIP1 and CDC37 in our MR analysis indicate that the genetic landscape of SSc is multifaceted. While RAC1 promotes disease, the downregulation or dysfunction of these protective genes may lower the threshold for disease onset. LRRFIP1, for example, is known to regulate cytosolic DNA sensing and Type‐I interferon production. Its protective role suggests that maintaining proper regulation of innate immune sensing is crucial for preventing the autoimmune cascade in SSc. The contrast between the risk effect of RAC1 and the protective effect of other markers provides human evidence that the balance between profibrotic signaling and immune‐regulatory mechanisms is dysregulated in SSc. The impaired function of these protective pathways may affect their ability to buffer against environmental triggers, thereby promoting the progression of SSc.

Our validation analysis using bulk RNA‐seq data from external cohorts corroborated the MR findings, showing that RAC1 expression is significantly elevated in SSc tissues compared to healthy controls (*p* = 3.7e‐05). This transcriptomic evidence aligns perfectly with the genetic prediction, reinforcing the conclusion that RAC1 is a pathogenic driver that is aberrantly activated in the disease context. Unlike the complex colocalization results often seen in GWAS studies where signals may diverge, the consistency between our eQTL‐based MR analysis and the actual differential expression in patient tissues suggests a robust biological effect. This indicates that the effect of RAC1 genetic variants on SSc risk likely operates directly through alterations in RAC1 expression levels, driving the downstream pathological phenotypes observed in pericytes.

The *RAC1* + IM pericyte cluster displayed a unique cellular interaction profile, engaging extensively with local immune and endothelial networks. Specifically, when juxtaposed with *RAC1*‐negative pericytes, this *RAC1*‐enriched subset exhibited significantly amplified signaling directed at monocytes and macrophages, heavily relying on the MIF‐(CD74/CD44) ligand‐receptor pair [27]. Consequently, we hypothesize that *RAC1* dictates local immunomodulation by triggering pericytic chemokine secretion (e.g., CCL2 and CXCL8). In parallel, the activation of the MIF‐(CD74+CD44) pathway on macrophages emerges as a critical driver exacerbating the disease progression. This axis is known to promote the recruitment of inflammatory cells and facilitate the survival of fibroblasts. Our findings suggest that RAC1+ pericytes do not merely contribute to the myofibroblast pool via transdifferentiation but also actively orchestrate the immune microenvironment. Additionally, our metabolic analysis revealed that this activated pericyte state is supported by distinct metabolic reprogramming, particularly the upregulation of riboflavin and thiamine metabolism. This bioenergetic shift likely provides the necessary substrates for the high demands of proliferation and cytokine secretion, creating a metabolic vulnerability that could be therapeutically exploited.

MR leverages the natural stochastic assortment of genetic alleles to deduce causal links between specific traits and disease susceptibility [28]. A primary strength of this approach lies in its robust mitigation of confounding variables; because genetic instruments are fixed at conception, the analysis is intrinsically protected against reverse causality [29]. Nevertheless, the methodology is not without constraints. Individual genetic polymorphisms typically exert only modest effects on target exposures, potentially compromising statistical power and elevating the probability of false‐negative outcomes [30, 31]. Consequently, integrating MR with orthogonal validation strategies and supplementary analytical frameworks is imperative to ensure robust and accurate conclusions [32]. In our study, we addressed this by integrating high‐resolution single‐cell transcriptomics with MR, providing a multidimensional validation of our findings [33, 34]. However, we must acknowledge that our conclusions regarding the distinct metabolic reprogramming in RAC1+ pericytes are derived solely from transcriptomic pathway enrichment. The absence of direct metabolomic validation constitutes a limitation of the current study, highlighting the need for targeted biochemical profiling in future investigations to confirm these specific bioenergetic shifts. Recognizing the exploratory nature of this study, the mechanistic links proposed herein are computationally derived, and their actual translational viability mandates prospective in vivo and clinical validation. Although our present analysis deliberately centered on the *RAC1*‐driven pericyte axis in SSc, the broader repertoire of dysregulated targets captured within our cohorts presents compelling, yet unexplored, avenues for future therapeutic discovery. Future studies should focus on validating the specific role of the RAC1‐MIF‐CD74 axis in animal models to confirm whether targeting this pathway can reverse established fibrosis [25].

In conclusion, our study provides a comprehensive overview of the cellular and genetic mechanisms linking pericyte dysfunction to SSc. By identifying the expansion of progenitor‐like pericytes and pinpointing RAC1 as a causal genetic driver, we offer new insights into the “vascular‐fibrotic” link. The revelation that RAC1 drives both structural remodeling and immune crosstalk via metabolic reprogramming opens new avenues for therapeutic intervention, potentially transforming the management of this debilitating disease [35–37]. Although direct RAC1 inhibitors remain primarily in preclinical development, our pharmacological enrichment analysis successfully identified several FDA‐approved drugs capable of potentially reversing the RAC1‐driven pathogenic signature in pericytes. Notably, rifampicin emerged as the top candidate, an agent increasingly recognized for its pleiotropic anti‐fibrotic properties. Furthermore, masitinib, a potent inhibitor of PDGFR—a central signaling hub in pericyte biology and SSc pathogenesis—was also highly enriched. These findings suggest that repurposing such existing FDA‐approved compounds could indirectly modulate the profibrotic networks downstream of the RAC1 axis, offering a readily translatable therapeutic avenue for SSc.

## 5. Conclusion

By cross‐examining scRNA‐seq profiles and bidirectional MR data, we successfully decoded the causal architectures and common pathological pathways linking pericyte dysregulation to SSc. A central finding within the pericyte population was the characterization of a unique progenitor‐like subset, which robustly proliferates under SSc conditions and actively commits to a fibrotic cellular fate [38]. Mechanistic exploration revealed that RAC1 is a causal risk factor that drives this pathological transition, promoting a proinflammatory state characterized by enhanced immune cell recruitment and distinct metabolic reprogramming [39]. By successfully bridging the molecular gap between RAC1− dependent pericyte dysregulation and SSc development, this study yields indispensable theoretical insights. Ultimately, unraveling this specific axis paves the way for innovating targeted therapeutic paradigms against RAC1 signaling [40].

## Author Contributions

**Xiaoqing Wang**: conceptualization, methodology, software, formal analysis, writing – original draft, visualization. **Zheng Zhang**: methodology, validation, formal analysis, investigation, data curation, writing – original draft. **Enze Jiang**: software, validation, resources, writing – review and editing. **Mengdi Zhang**: conceptualization, resources, supervision, project administration, writing – review and editing. **Guanglei Hu**: conceptualization, methodology, supervision, funding acquisition, writing – review and editing.

## Funding

This study was funded by the National Natural Science Foundation of China (Grant 32400808).

## Disclosure

This study used only publicly available datasets and Mendelian randomization analyses, and no cell lines were used. Therefore, the journal’s cell line authentication policy is not applicable.

## Conflicts of Interest

The authors declare no conflicts of interest.

## Supporting Information

Additional supporting information can be found online in the Supporting Information section.

## Supporting information


**Supporting Information 1** Figure S1: This figure demonstrates the significant downregulation of the causally protective genes, LRRFIP1 and CDC37, in the systemic sclerosis single‐cell dataset compared to healthy controls.

**Supporting Information 2** Table S1: This table provides a list of FDA‐approved compounds identified via Connectivity Map (CMap) analysis that are capable of potentially reversing the RAC1+ profibrotic transcriptomic signature.

## Data Availability

The single‐cell RNA sequencing data used in this study are publicly available in the Gene Expression Omnibus database under Accession Number GSE138669. The bulk RNA‐seq validation datasets are available in the GEO database under Accession Number GSE130955. Summary statistics for the genome‐wide association study of systemic sclerosis were obtained from the FinnGen consortium (ID: finn‐b‐M13_SYSTSLCE).
