# Supplementary material for: Integrating Single‐Cell Transcriptomics and Mendelian Randomization to Identify RAC1 as a Causal Metabolic Driver of Pericyte Dysfunction in Systemic Sclerosis
Source: Mediators Inflamm. 2026 Jun 16;2026:6166654. doi: 10.1155/mi/6166654 (PMC13270254; doi:10.1155/mi/6166654)
Supplement: Supplementary file 1 — Supporting Information 1 Figure S1: This figure demonstrates the significant downregulation of the causally protective genes, LRRFIP1 and CDC37, in the systemic sclerosis single‐cell dataset compared to healthy controls. [file MI-2026-6166654-s001.docx]

**Differential expression of CDC37 and LRRFIP1 in Systemic Sclerosis (SSc) patients and healthy controls**


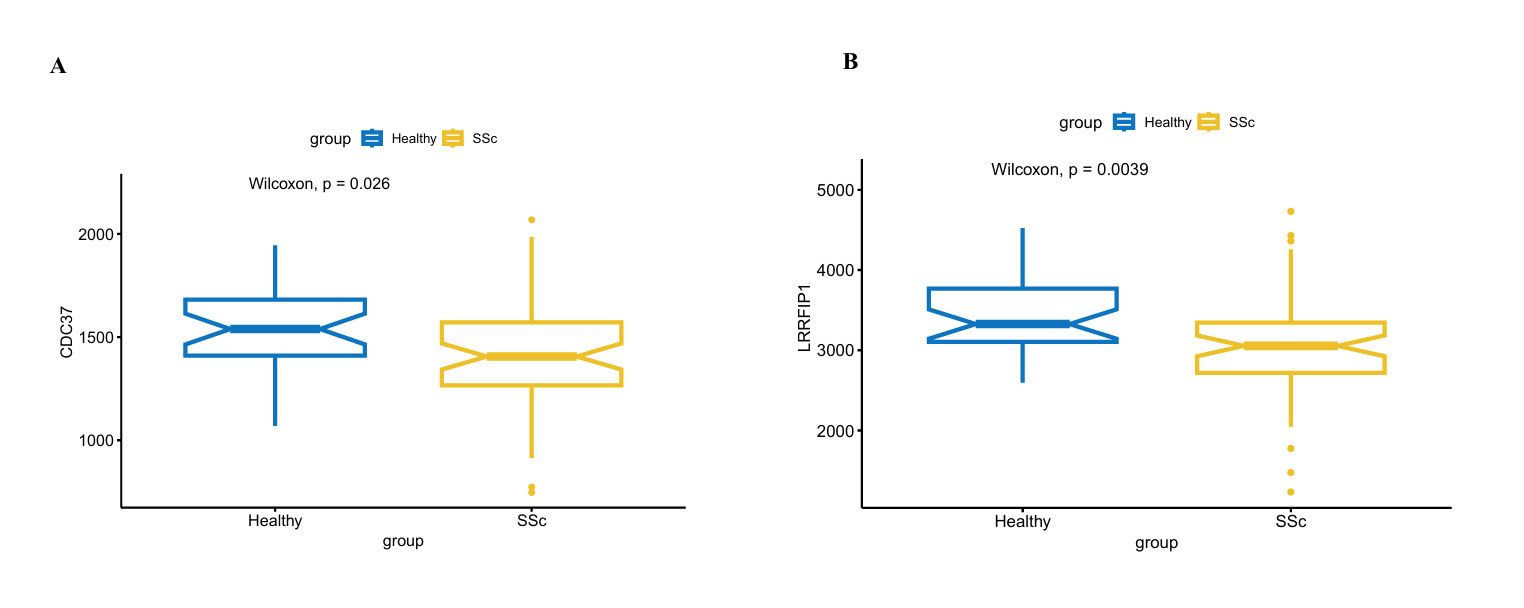
**Figure Legend**

**(A)** Boxplot illustrating the expression levels of CDC37 in the healthy control group (Healthy, blue) and the systemic sclerosis group (SSc, yellow). Statistical significance between the two groups was determined using the Wilcoxon rank-sum test (*p* = 0.026). **(B)** Boxplot comparing the expression levels of LRRFIP1 between the healthy control and SSc groups (Wilcoxon rank-sum test, *p* = 0.0039).
